# Rainfall affects leaching of pre-emergent herbicide from wheat residue into the soil

**DOI:** 10.1371/journal.pone.0210219

**Published:** 2019-02-01

**Authors:** Yaseen Khalil, Ken Flower, Kadambot H. M. Siddique, Phil Ward

**Affiliations:** 1 School of Agriculture and Environment, The University of Western Australia, Perth, WA, Australia; 2 The UWA Institute of Agriculture, The University of Western Australia, Perth, WA, Australia; 3 CSIRO, Wembley, WA, Australia; Duzce Universitesi, TURKEY

## Abstract

No-tillage with stubble retention is a widely used cropping system for its conservation and yield benefits. The no-tillage farming system in southern Australia relies heavily on herbicides for weed management, but heavy crop residues may have a negative impact on the activity of pre-emergent herbicides applied. Any herbicide intercepted by the crop residue may not reach the soil surface without timely rainfall and may dissipate due to volatilisation, photo-degradation and/or microbial activity. Two experiments were carried out to investigate the interception of prosulfocarb, pyroxasulfone, and trifluralin herbicides by wheat residue and retention following simulated rainfall. For the first experiment, there were four simulated rainfall amounts (0, 5, 10, and 20 mm), three intensities (5, 10, and 20 mm h^–1^) and five application times (immediately after spraying herbicide, 6 h, 1, 7, and 14 days after spraying). In the second experiment, 20 mm of rainfall was applied at 10 mm h^–1^ in either 4 × 5 mm rainfall events over two days, 2 × 10 mm rainfall events over one day, or a single 20 mm rainfall event, with a no-rainfall control treatment. Bioassays were used to assess the herbicide activity/availability in the soil and remaining on the residue, using cucumber (*Cucumis sativus* L.) and Italian ryegrass (*Lolium multiflorum* Lam.) as indicator plants. At higher rainfall amounts, most of the herbicide leached from the stubble into the soil soon after application; more so with rain in one event rather than multiple events. However, the intensity of rainfall had no effect. Pyroxasulfone leached easily from the residue to the soil to potentially offer good weed control, prosulfocarb had an intermediary leaching effect, while only a small amount of trifluralin leached from stubble after rain. Therefore, in no-tillage situations with large amounts of crop residue present on the soil surface, herbicides that leach easily from the residue should be considered, like pyroxasulfone.

## Introduction

Conservation agriculture, with minimal or no soil tillage and maximum residue retention, known as no-tillage (NT), is widely practised in Australia and many regions around the world [1–3]. The NT system is heavily dependent on herbicides for weed control [4], but heavy crop residues may have a negative impact on the activity of these herbicides [5]. Herbicides can be intercepted by the crop residue [6–9] and may not reach the soil surface without timely rainfall to wash them off the crop residues into the soil below, where they will be available to control germinating weeds [5, 6, 10–13]. For example, crop residues have been shown to intercept 15–80% of the applied herbicide, which may account for the reduction in herbicide effectiveness sometimes observed in NT systems [8]. The herbicide remaining on the residue may also dissipate due to volatilisation, photo-degradation and/or microbial degradation [14, 15]. The rainfall amount and intensity can significantly impact herbicide wash-off from residue and its movement and dissipation in NT soils [16–19]. On the other hand, a number of studies reported that intensity of rain does not affect the amount of herbicide washed off, rather, the total amount of rain is important, but probably does influence movement but maybe not dissipation [20–22]. In addition, the frequency of rainfall events affects moisture levels [23] and the variations in drying and wetting periods influence microbial processes occurring at the residue–soil interface [24]. Westra et al. [25] showed that the dissipation half-life of pyroxasulfone (5-(difluoromethoxy)-1-methyl-3-(trifluoromethyl) pyrazol-4-ylmethyl 4,5-dihydro-5,5-dimethyl-1,2-oxazol-3-yl sulfone) ranged from 47 to 134 days and appeared to be influenced by soil type and the movement of herbicides below 30 cm in the soil following rainfall. The mobility of the herbicide was dependent on the site–year conditions with pyroxasulfone moving further down the soil profile than *S*-metolachlor (2-chloro-*N*-(6-ethyl-*o*-tolyl)-*N*-[(1*RS*)-2-methoxy-1-methylethyl] acetamide).

Pre-emergent herbicides are intended to be applied to the soil, and many require incorporation by rainfall, irrigation, tillage or, in the case of NT systems, the seeding operation [26–28]. The activity of pre-emergent herbicides applied to crop residues depends not only on the physicochemical properties of the herbicides, but the amount and origin of the crop residues, spray volume, and the period prior to the first rainfall event after application and the duration of following rainfall events [29–31]. A study on the transfer of atrazine (6-chloro-*N*^2^-ethyl-*N*4-isopropyl-1,3,5-triazine-2,4-diamine) from standing and flat wheat stubble showed that, three weeks after application and 50 mm of rainfall, atrazine declined by 90% on standing residue and 63% on flat residue, while the amount of herbicide in the soil doubled. Nine weeks after application, there was no atrazine left on the residue [6]. Williams and Wicks [32] showed that 70% of the applied atrazine reached the soil surface within the initial 90 days in an NT system when 85% of the soil surface was covered by crop residues. For pre-emergent herbicides, such as atrazine, pyroxasulfone, and metolachlor, most of the chemicals will wash off the residue with as little as 5 mm of rainfall [13, 33, 34]. However, differences exist depending on the type of crop residue and the herbicide. Some herbicides, such as metolachlor, can volatilise from the crop residue if rainfall does not occur soon after application [13, 33–36].

Commonly used pre-emergent herbicides for weed control in southern Australia are prosulfocarb (S-benzyl dipropyl(thiocarbamate)), pyroxasulfone and trifluralin (α,α,α-trifluoro-2,6-dinitro-*N*,*N*-dipropyl-*p*-toluidine). This region has a Mediterranean-type climate, with mild, wet winters and hot, dry summers. NT with crop residue retention is the predominant cropping system [35, 36] with pre-emergent herbicides applied and then thrown, with associated soil, out of the crop row by the seeding machinery, thereby reducing or avoiding phytotoxicity. The small amount of soil thrown out of the crop row also covers/incorporates herbicide in the inter-row. Many farmers now start seeding prior to seasonal rain under dry soil conditions (sometimes called dry-seeding) [34], which may affect the efficacy of the herbicides. Therefore, it is important to understand the longevity of pre-emergent herbicides on stubble and their interaction with rainfall. The main objectives of this research were to determine (1) the effect of rainfall timing, amount and intensity and (2) the effect of single and multiple rainfall events on leaching of prosulfocarb, pyroxasulfone and trifluralin from wheat stubble.

## Materials and methods

Two experiments were conducted to determine the effect of rainfall on leaching of prosulfocarb, pyroxasulfone and trifluralin from wheat stubble. The first experiment focused on rainfall amount, intensity and timing, while the second compared single and multiple rainfall events. Briefly, the herbicides were sprayed onto plastic trays containing soil in Petri dishes that were covered with wheat residue. Simulated rainfall, which is explained below in more detail below, was then applied to the trays, and the amount of herbicide remaining in the residue and leached into the soil was determined by bioassays.

### Soil and crop residue

The soil was typical of the Western Australian wheatbelt and was collected from the surface (0–10 cm) of a farm paddock in the Cunderdin area of Western Australia (–31.58442° S, 117.327038° E), after obtaining permission from the farm owner. The soil was air-dried and passed through a 2-mm sieve, with a sample analysed for texture, pH (CaCl_2_), CEC and organic carbon, using the methods of [37], the Soil Science Laboratories of University of Western Australia, Perth, and the CSBP Soil and Plant Laboratory (www.csbp-fertilisers.com.au). The soil was acidic and classified as a sandy loam (Table 1).

**Table 1:** Properties of the soil used in the bioassays.

pH	OM	CEC	Al	Ca	K	Mg	Na	Sand	Silt	Clay
(CaCl_2_)	(%)	(cmol(+)kg^–1^)						(%)	(%)	(%)
4.4	1.8	2.96	0.02	2.75	0.14	0.45	0.07	74	12	14

Dry wheat stubble was collected shortly after harvest from the same Cunderdin field as the soil. After being sprayed with herbicide (described below) and the various rainfall applications, the wheat residue was then air-dried, ground into small particles using a mechanical plant material grinder (www.retsch.com), and then used as one of the germination media in the bioassay. After grinding, the length of about 85% of the particles ranged from 1–4 mm. To minimise sample contamination, the grinder was cleaned after each batch, using a vacuum and then air compressor (forced-air blower).

### Herbicide application

Commercial formulations of the three pre-emergent herbicides were applied at recommended field rates: prosulfocarb 2000 g a.i. ha^–1^, pyroxasulfone 102 g a.i. ha^–1^ and trifluralin 960 g a.i. ha^–1^ [38]. The herbicides were applied using a twin-nozzle laboratory sprayer fitted with 110° 01 flat-fan spray jets (Tee jet) delivering 117.1 L ha^–1^ at 210 kPa, travelling at a speed of 3.6 km h^–1^. Selected properties of herbicides selected (Table 2).

**Table 2:** Vapour pressure, solubility, average adsorption coefficients, and average DT_50_ values for herbicide studied [39].

Pre-emergent herbicide	Prosulfocarb	Pyroxasulfone	Trifluralin
**Vapour pressure (mPa @ 25°C)**	0.79	2.4×10^−3^	9.5
**Solubility (mg L^–1^ @ 20°C)**	3.5	5	0.22
**Average K_oc_ value^**	95	130	15800
**Average DT50 value^**	22 (16–26)	90	170 (35–375)

### Rainfall simulation set up and calibration

The rainfall simulator was based on a design used by Meyer [40] and Hermsmeier et al. [41]. Four pillar legs supported the rainfall simulator, which was 2.4 m above the ground and consisted of a metal frame shaped in a truncated pyramid (3.10 × 2.70 m at the base, 3.0 × 0.26 m at the top, 2.5 m high). The simulated rainfall was applied through three VeeJet flat-fan nozzles (Spraying Systems, Wheaton, IL) that were turned back and forth through 45° by a motor, at a pre-determined rate. A pressure gauge on top of the water inlet manifold was used to regulate the flow of water to the nozzles, and the wait-time of the nozzles at the end-point could be altered to vary the rainfall application rate. Three nozzle types were used to achieve the three different rainfall intensities of 5, 10 and 20 mm h^−1^ (VeeJet 8020, 8050, and 80100) using a constant pressure of 80 KPa. The rainfall simulator was calibrated prior to the experiment by placing four rain gauges on the ground below the nozzles to catch the rainfall and to determine the resulting rainfall amount (mm) and intensity (mm h^–1^). The process was repeated until the desired rainfall amounts and intensities were achieved.

### Rainfall treatments

The trials were conducted at The University of Western Australia School of Agriculture and Environment facilities (–31.9812° S, 115.8199° E). Four Petri dishes, each representing one replication and containing 50 g of dry soil, were placed onto plastic seedling trays; the trays and Petri dishes were uniformly covered with 39 g wheat residue (an equivalent of 4 t ha^–1^ wheat residue, which is the average amount expected to be found in the field) and sprayed with one of the three herbicides at the recommended field rate or not sprayed (untreated control). In the first experiment, simulated rainfall was applied in one of four amounts (0, 5, 10, or 20 mm), three different intensities (5, 10, or 20 m h^–1^) and five application times after spraying the herbicide (immediately after spraying (0), 0.25 (6 hours), 1, 7, or 14 days). In the second experiment, the herbicides were applied over the residue and this was immediately followed by four rainfall treatments arranged in a randomised block with four replications: (1) no rainfall, or 20 mm of rainfall applied as (2) 4 × 5 mm rainfall events over two days, (3) 2 × 10 mm rainfall events over one day, or (4) a single 20 mm rainfall event. For the second experiment, the rainfall treatments were applied at an intensity of 10 mm h^–1^.

After spraying, the Petri dishes with soil were placed in a –20°C freezer prior to the bioassays. The wheat residue was air-dried and ground, as previously described, and placed in plastic bags and stored at –20°C prior to the bioassays.

### Bioassay conditions

The method for the bioassays was previously described by Khalil et al. [42]. Briefly, the bioassays were conducted in a growth room on shelves equipped with LUMILUX cool white fluorescent lamps (Model L36W/840, OSRAM), with photosynthetically active radiation (PAR) at the top of the plants of 109 μmol m^–2^ s^–1^ (SD ±5 μmol m^–2^ s^–1^), and a 12-hour photoperiod. The air temperature was maintained at 25/22.5°C (SD ±2/1°C) during the light/dark period. Relative humidity in the room was 70% (SD±10%). Petri dishes (9 cm diameter) were filled with either 50 g soil or 5 g ground wheat residue, and five seeds of Italian ryegrass (*Lolium multiflorum* Lam., Dargo, Irwin Hunter Seeds, Unit11, 88 Forrest St, Cottesloe, WA 6011, www.irwinhunter.com.au) and cucumber (*Cucumis sativus* L., Long Green Supermarket), Mr. Fothergill’s Seeds, 15B Walker St, South Windsor NSW 2756, www.mrfothergills.com.au) were planted in the same Petri dish at 1 cm depth. The plants were hand watered on a daily basis by adjusting the moisture of the medium to near field capacity with deionised water [43]. After seven days, the media was washed from the plants with running tap water and the plants removed for shoot length measurements. The percent shoot length inhibition from the untreated control (UTC) was calculated for each media using the formula [*shoot length (% of untreated control) = L*_*t*_ *× (100/L*_*0*_*)*], where L_t_ is the shoot length measured in the herbicide-treated soil or wheat residue, and L_0_ is the shoot length in the untreated soil or wheat residue. Shoot length of the bioassay plant species was used as an indicator of herbicide concentration in the media, with greater shoot length equating to lower herbicide concentration.

### Data analysis

The germination of ryegrass was >98% and cucumber 100% in the untreated controls. Each herbicide and bioassay species were analysed separately. Both ryegrass and cucumber data are shown, as in some instances no ryegrass germinated, especially with pyroxasulfone, and in these situations the data was not included in the analysis. All cucumber seeds germinated for all herbicide treatments. The data were tested for normality and homogeneity of variance before conducting ANOVA on the shoot length data, using GenStat 12 [44], to test for significance at P ≤ 0.05.

For the first experiment, a three-way ANOVA was performed (rainfall intensity × rainfall amount × rainfall timing) and a one-way ANOVA for the second experiment, to compare the four treatments.

## Results and discussion

**Fig 1:**
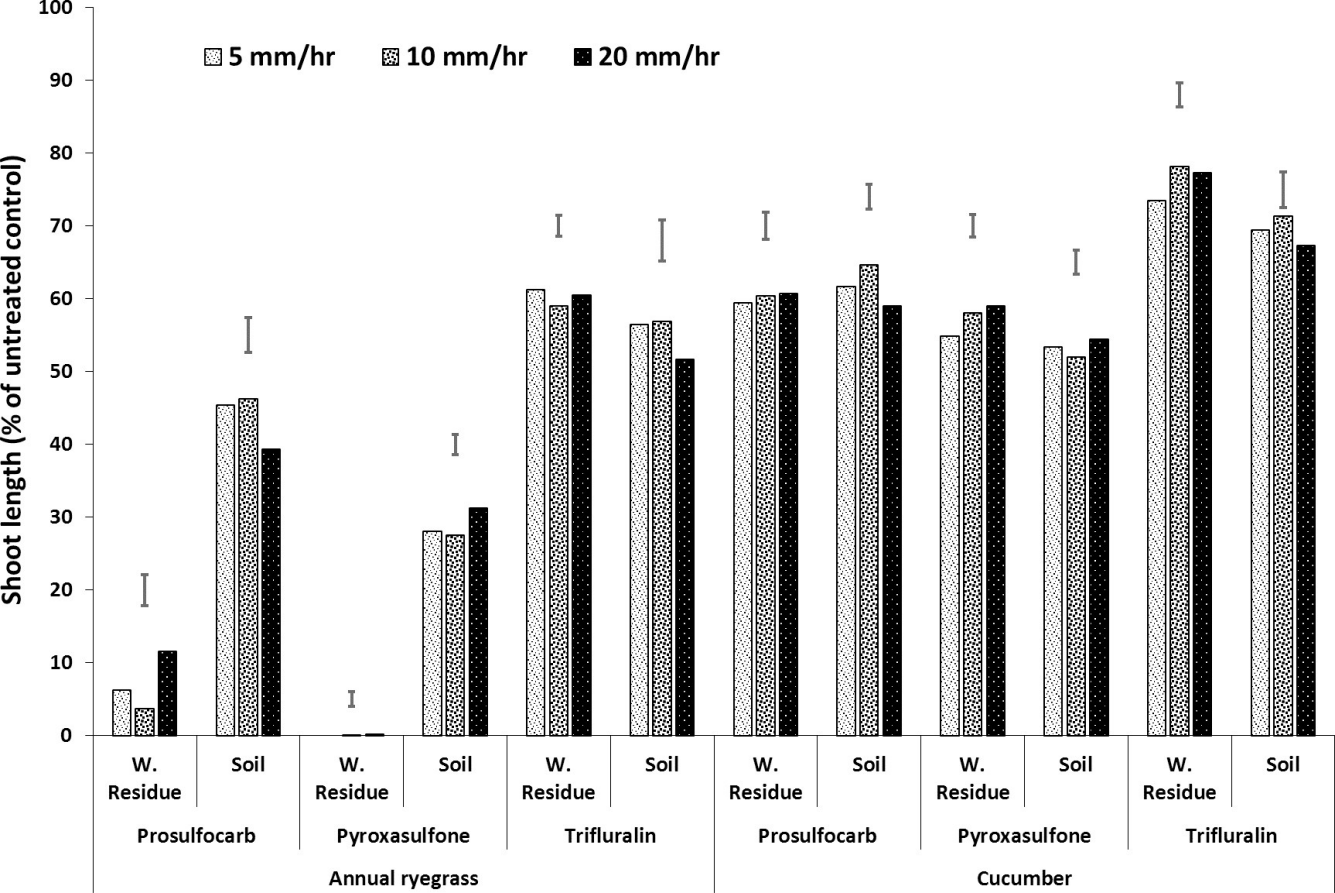
Shoot length (% of untreated control) of bioassay species (Italian ryegrass and cucumber) grown in wheat residue and soil after spraying prosulfocarb, pyroxasulfone and trifluralin followed by different rainfall intensities (5, 10 and 20 mm h^–1^). Bars show LSD at P = 0.05 for comparisons within rainfall intensities for each media and herbicide, where significant differences were found.

**Fig 2:**
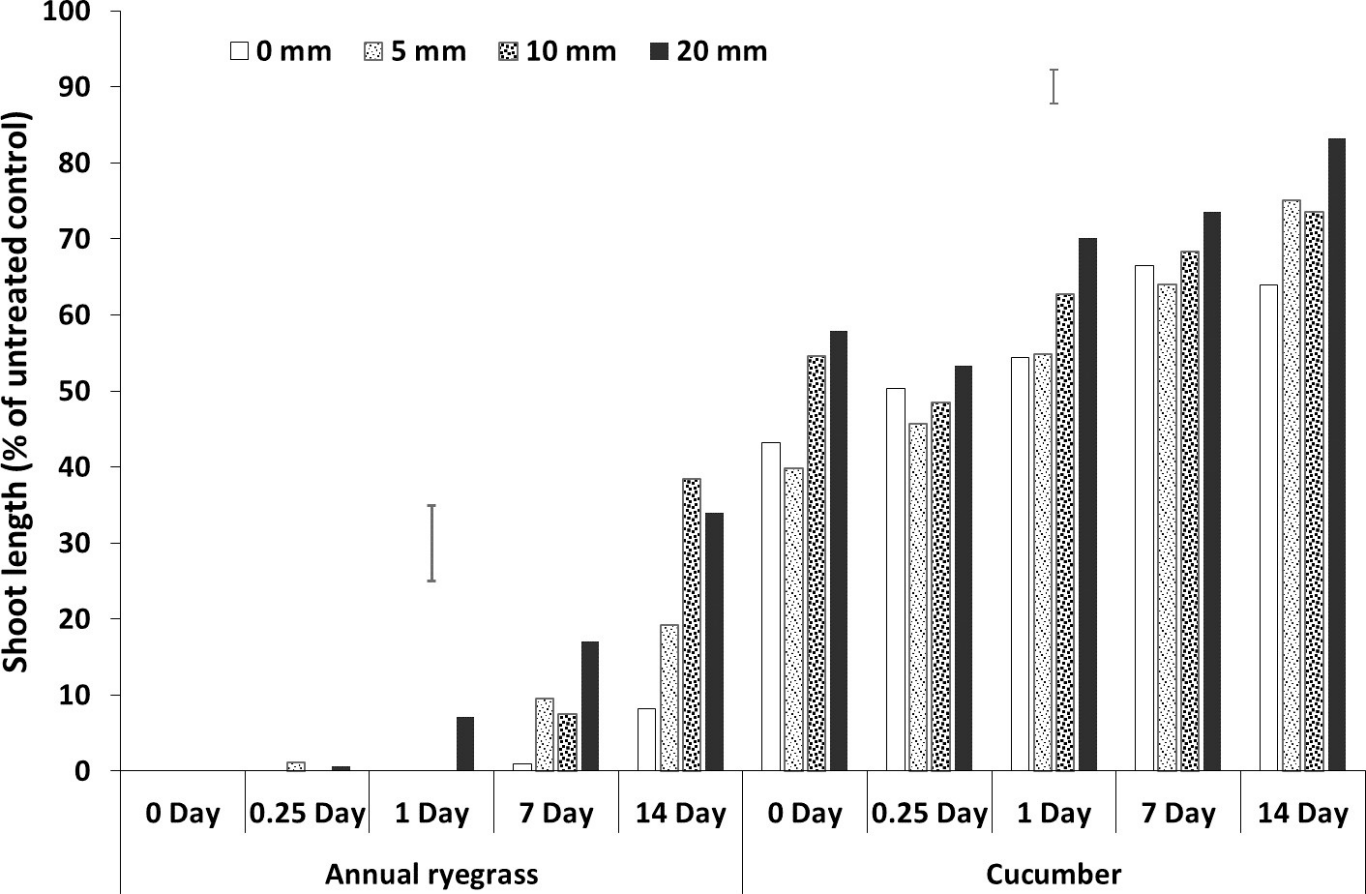
Shoot length (% of untreated control) of bioassay species grown in wheat residue after spraying prosulfocarb followed by different amounts and timing of rainfall. Bars show LSD at P = 0.05 for comparisons within each bioassay species.

**Fig 3:**
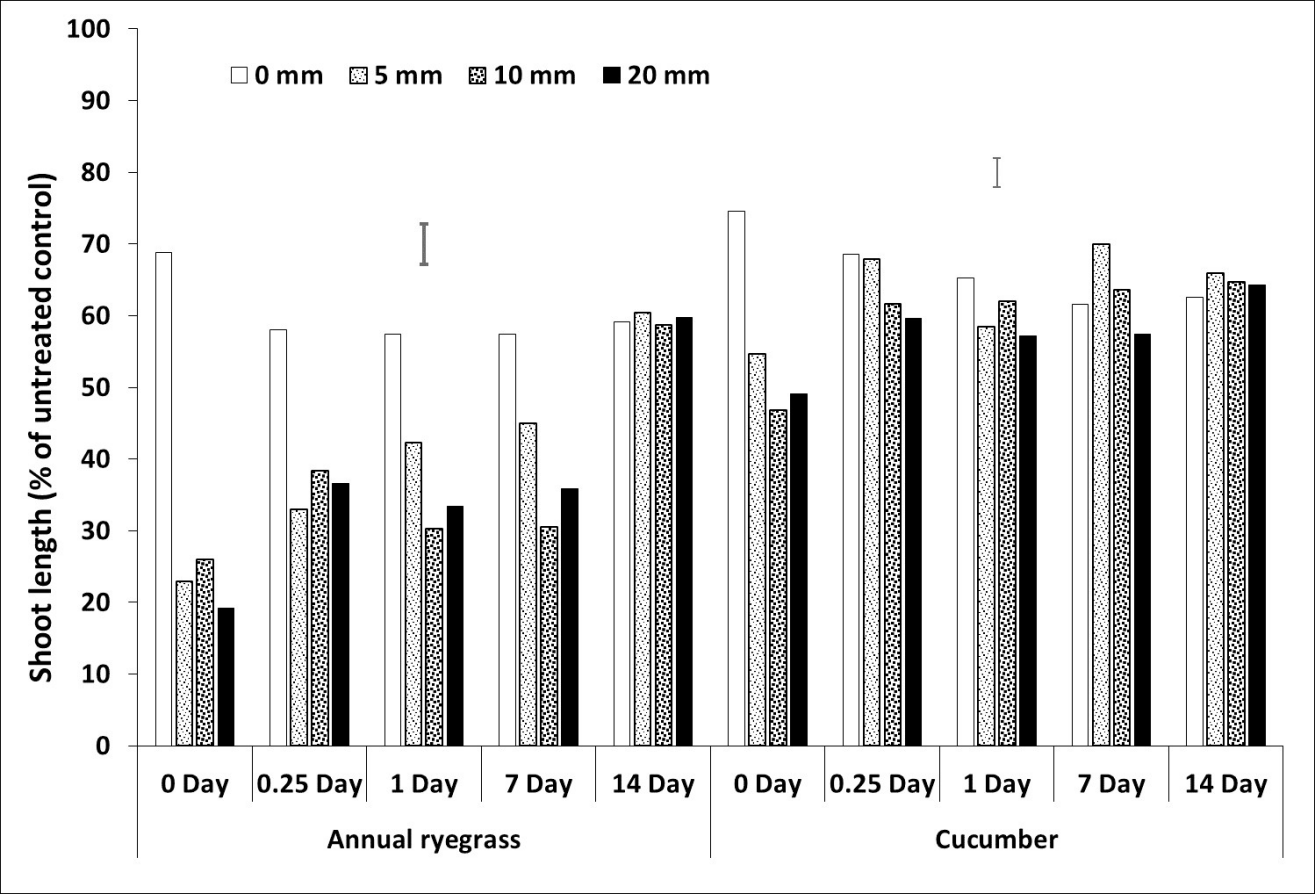
Shoot length (% of untreated control) of bioassay species grown in soil after spraying prosulfocarb followed by different amounts and timing of rainfall. Bars show LSD at P = 0.05 for comparisons within each bioassay species.

**Fig 4:**
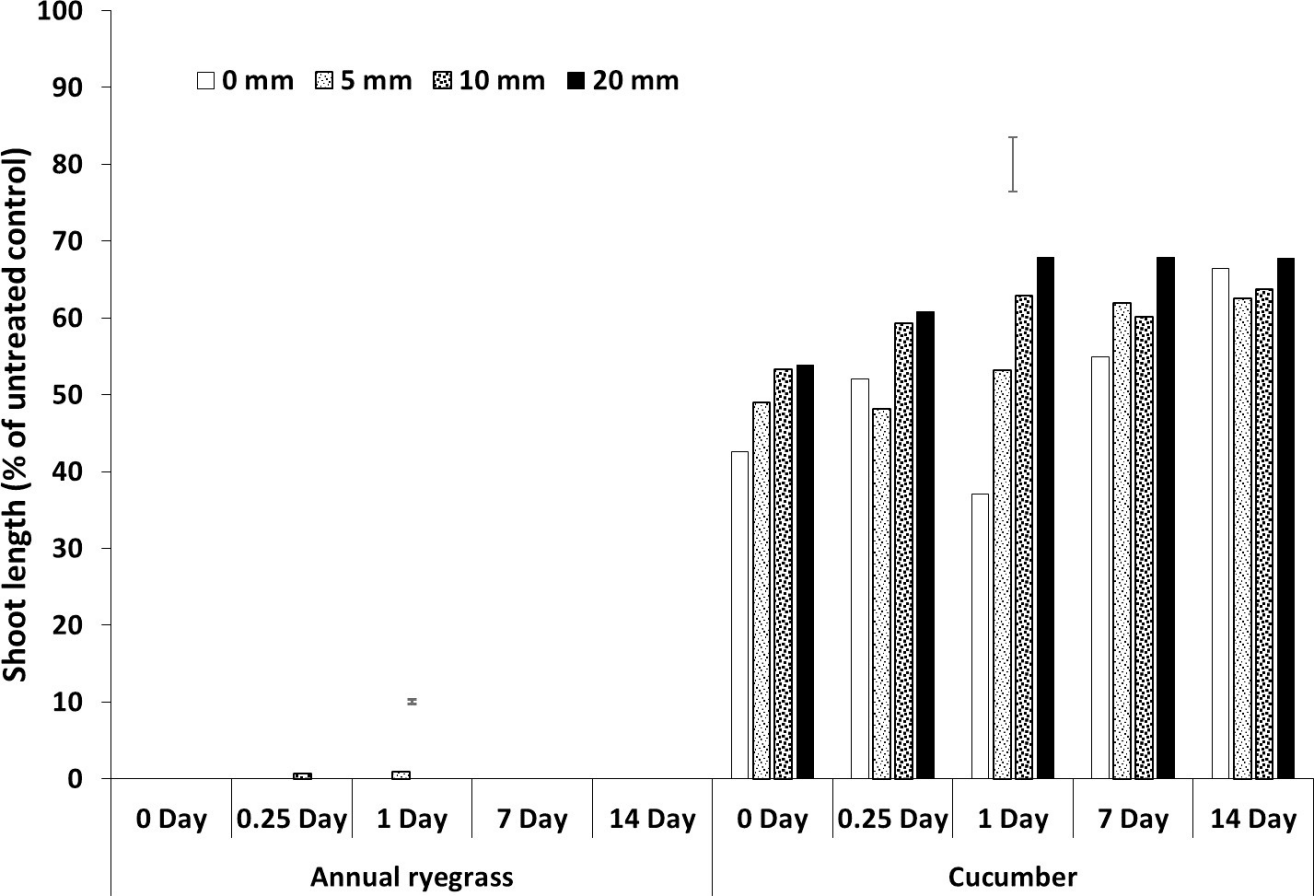
Shoot length (% of untreated control) of bioassay species grown in wheat residue after spraying pyroxasulfone followed by different amounts and timing of rainfall. Bars show LSD at P = 0.05 for comparisons within each bioassay species.

**Fig 5:**
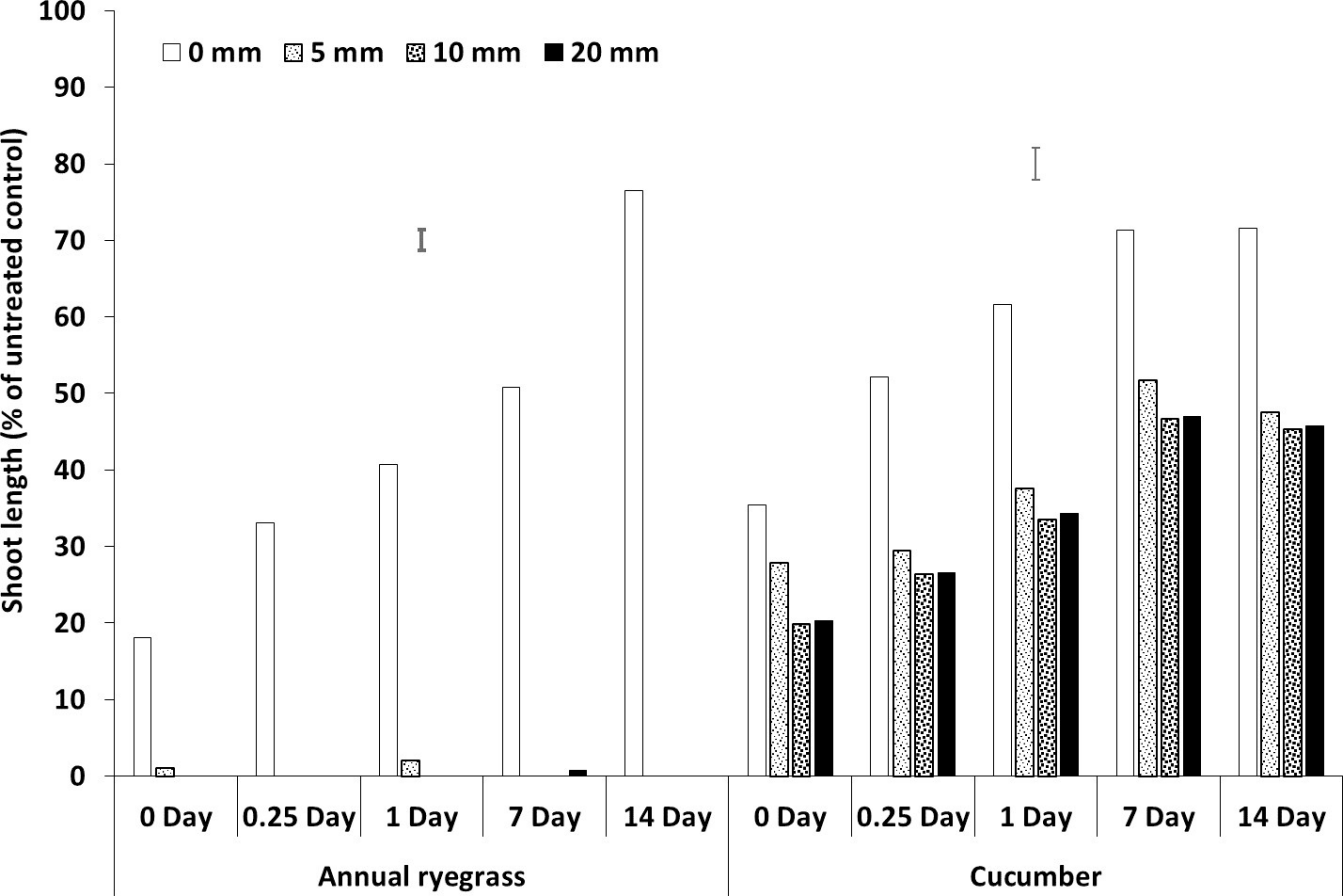
Shoot length (% of untreated control) of bioassay species grown in soil after spraying pyroxasulfone followed by different amounts and timing of rainfall. Bars show LSD at P = 0.05 for comparisons within each bioassay species.

**Fig 6:**
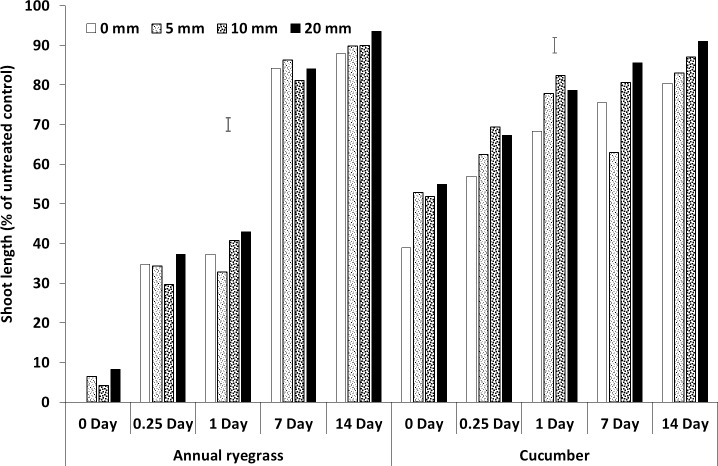
Shoot length (% of untreated control) of bioassay species grown in wheat residue after spraying trifluralin followed by different amounts and timing of rainfall. Bars show LSD at P = 0.05 for comparisons within each bioassay species.

**Fig 7:**
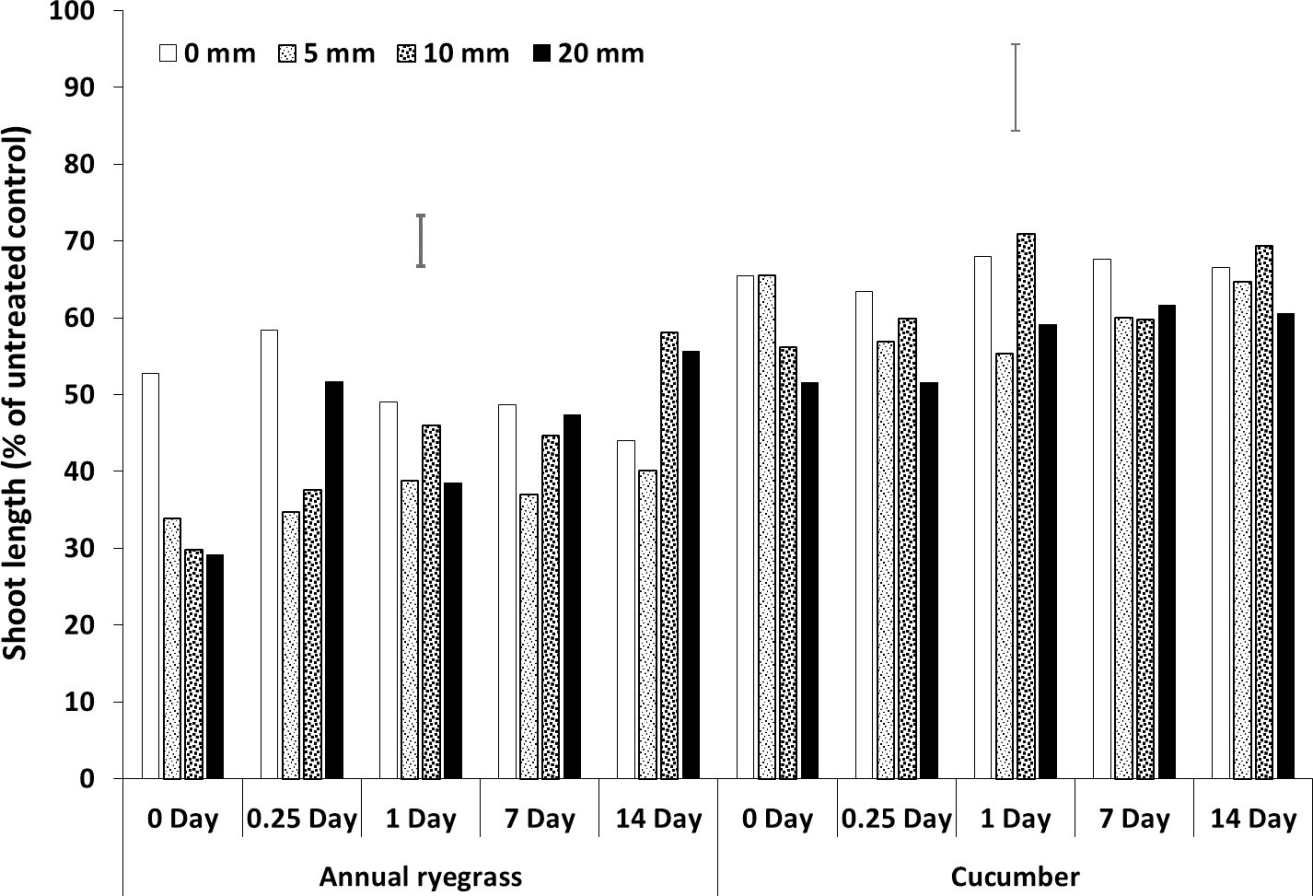
Shoot length (% of untreated control) of bioassay species grown in soil after spraying trifluralin followed by different amounts and timing of rainfall. Bars show LSD at P = 0.05 for comparisons within each bioassay species.

Rainfall generally leached the herbicides from the residue into the soil, resulting in increased shoot growth of the bioassay species in the residue and a corresponding decrease in shoot growth in the soil, as the concentrations of herbicide increased. Overall, annual ryegrass was more sensitive to the three herbicides than cucumber, particularly for pyroxasulfone (Figs 1–7, S1 Table, S1 Text). In the first experiment, there were no interactions between rainfall intensity and rainfall amount or rainfall timing, so the mean results for each rainfall intensity are shown (Fig 1, S1 Table, S1 Text). There was a significant interaction between rainfall amount and rainfall timing on the leaching of intercepted herbicides from wheat residue (Figs 2–7, S1 Table, S1 Text).

### Rainfall intensity

Rainfall intensity had no consistent effect on the leaching of herbicide from the residue into the soil, although there was a trend for the intensity of 20 mm h^–1^ to leach more prosulfocarb and trifluralin into the soil than the lower intensities, but not for pyroxasulfone (Fig 1, S1 Table, S1 Text). Also, there were more phytotoxic levels of herbicide (i.e. reduced shoot length) in the residue compared with the soil for all intensities for both prosulfocarb and pyroxasulfone, but not for trifluralin. Varying the intensity of rainfall between 5 mm h^–1^ and 20 mm h^–1^ had minor and inconsistent effects on herbicide leaching from the residue to the soil. Willis et al. [21, 22] and McDowell et al. [20] also found that wash-off of insecticides from plant canopies depended on rainfall amount and was not affected by rainfall intensity.

### Rainfall amount and timing

All three herbicides had interactions between rainfall amount and timing (Figs 2–7, S1 Table, S1 Text). For prosulfocarb, higher amounts of rainfall leached more herbicide from the residue, resulting in increased growth of the bioassay plants in the residue, even after 14 days. There was a corresponding reduction in bioassay plant growth in the soil, except at 14 days, when there was no difference between the rainfall amounts, other than no-rainfall (Figs 2 and 3, S1 Table, S1 Text). Leaching of pyroxasulfone from the residue followed a similar pattern to prosulfocarb, except that few ryegrass germinated at any rainfall amount after 14 days, showing the sensitivity of this species to pyroxasulfone and how easily this herbicide washes of wheat residue into the soil. There was a corresponding reduction in bioassay plant growth in the soil with rainfall amount, which still occurred with rainfall 14 days after herbicide application (Figs 4 and 5, S1 Table, S1 Text).

Much of the sprayed herbicide was intercepted by the crop residue as seen in the reduced shoot length in the residue compared with the soil. This was also clearly demonstrated by the shoot lengths for pyroxasulfone when no simulated rainfall was applied (Figs 4 and 5, S1 Table, S1 Text). This is particularly so as Khalil et al. [42] showed that the ED_50_ for these bioassay species in the soil was markedly less than in the residue by between 17-fold (pyroxasulfone) and 150-fold (trifluralin), therefore much more herbicide was required to reduce shoot length in residue than soil. Also, differences in rainfall amounts from 5 to 20 mm were not as obvious with pyroxasulfone as with prosulfocarb, indicating that the former was easily leached. For trifluralin, the ryegrass data showed that rainfall had no effect on herbicide leaching from the residue beyond one day after herbicide application. The cucumber data indicated that increasing amounts of rainfall tended to leach more herbicide from the residue for up to 14 days, although differences were small compared to the other herbicides. The soil data (shoot length of bioassay plant species) for trifluralin was more variable, but there was little additional leached chemical in the soil with rainfall after about one day after herbicide application (Figs 6 and 7, S1 Table, S1 Text).

The herbicide sprayed on the wheat residue lost efficacy over time, as shown by increased shoot length, and this loss could be due to degradation, photolysis or volatilization; although pyroxasulfone appeared to change the least. Trifluralin on the wheat residue appeared to have fully disappeared by 14 days after application (Figs 2–6, S1 Table, S1 Text), whereas the other two herbicides were still bioactive on the residue. Grass et al. [45] and Bedos et al. [46] reported that up to 96% of trifluralin was volatilised within the first 48 h following herbicide application. Generally, increasing amounts of rainfall from 5 to 20 mm applied soon after spraying leached more herbicide from the wheat residue, although the corresponding decrease in shoot growth in the soil was not as evident and changed over time. Pyroxasulfone seemed to leach well with as little as 5 mm of rainfall, but prosulfocarb generally required more rainfall to leach herbicide into the soil. Rainfall reduced shoot length in the soil up to 7 days after application with prosulfocarb and up to 14 days with pyroxasulfone. No rainfall was applied beyond 14 days and it is possible that significant amounts of pyroxasulfone could still be washed from residue into the soil after this period. There appeared to be little trifluralin leached into the soil with rainfall one day after spraying. Similarly, Carbonari et al. [5] reported that the first 20 mm of simulated rainfall was responsible for washing off most of the sulfentrazone sprayed onto sugarcane residue. Carbonari et al. (2016) concluded that time of rainfall occurrence was a crucial factor affecting the availability of herbicides applied to crop residue. Similarly, Granovsky et al. [47] reported that a strong rainfall event soon after atrazine application washed off more of the chemicals intercepted by crop residues into the soil.

Bedos et al. [46] showed that volatilisation of trifluralin was very high immediately after application, but it declined in the following 24 h. The efficacy of trifluralin dropped by 62% by delaying incorporation for 48 h after application due to surface volatilisation [45]. Moreover, Haskins [28] and Selim et al. [9] reported that crop residues on the soil surface would inert some herbicides, such as trifluralin, and he recommended using higher label rates of these herbicides in conservation farming systems.

Despite trifluralin having the longest half-life of the three herbicides (35–375 days), it is subject to volatilisation and photo-degradation, especially with surface application without incorporation. The half-life of pyroxasulfone and prosulfocarb are lower (16–90 days) [28, 48]. Nonetheless, pyroxasulfone appeared the best of the three herbicides tested for conservation farming systems, especially for high residue situations, as a significant amount of herbicide leached from the residue with rainfall 14 days after application. Prosulfocarb was intermediate between pyroxasulfone and trifluralin, as some herbicide leached into the soil with rainfall after seven days. Further studies should be conducted to determine how much longer pyroxasulfone will leach from crop residue into the soil.

### Multiple rainfall events

**Fig 8:**
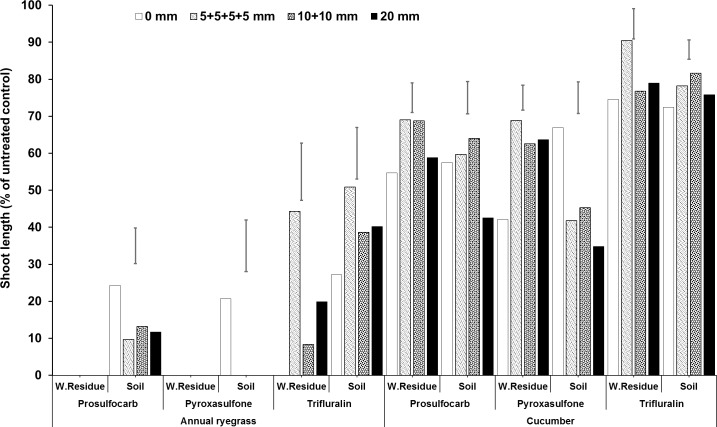
Shoot length (% of untreated control) of bioassay species (Italian ryegrass and cucumber) grown in wheat residue and soil after spraying prosulfocarb, pyroxasulfone and trifluralin followed by different rainfall treatments. Bars show LSD at P = 0.05 for comparisons within rainfall treatments for each medium, herbicide and bioassay plant species, where significant differences were found.

All rainfall treatments (≥5 mm) leached some prosulfocarb, pyroxasulfone and trifluralin from residue as seen in the increased shoot length in the residue bioassay compared with nil rainfall (Fig 8, S2 Table, S2 Text). Nonetheless, the residue remained phytotoxic to ryegrass for prosulfocarb and pyroxasulfone. Applying 20 mm of rainfall over the residue in a single event immediately after herbicide application generally gave better control (reduced shoot length) of the bioassay species in the soil than applying the same amount of rainfall in multiple events (4 × 5 mm events over two days or 2 × 10 mm events over one day), although the differences were relatively small (Fig 8, S2 Table, S2 Text). Therefore, even 5 mm of rainfall can effectively leach herbicide into the soil. For trifluralin, rainfall over the residue after spraying had little beneficial effect for controlling the plants growing in the soil below. For prosulfocarb with ryegrass, all three rainfall amounts reduced ryegrass shoot growth compared with 0 mm, while for cucumber only the 20 mm treatment differed significantly, with reduced shoot growth in the soil (Fig 8, S2 Table, S2 Text). It would be expected that shoot growth in the residue would be greater with 20 mm of rainfall applied as a single event, as more herbicide leached into the soil; however, in many instances, this was not the case. This can be explained by the effect of a longer period of wet residue, with the multiple rainfall events, which may have increased the loss of herbicide. Aslam et al. [15] showed that under a light and frequent rainfall regime, S-metolachlor dissipation in crop residues was quicker than a heavy and infrequent rain regime. This was due to wetter surface conditions, where crop residue decomposition was also faster. Microbial decomposition of crop residues is highly affected by water dynamics (rainfall) and temperature at the soil–residue interface [24, 49–51]. Unger [52] reported that crop residues that are often wet have low sorption capacity for pesticides and are less aerodynamically stable than the soil underneath, therefore have greater potential to dissipate.

## Conclusions

Some herbicide leached from the residue with as little as 5 mm of rainfall, although higher rainfall amounts generally leached more herbicide from the residue. The sooner the rainfall occurred, the greater the amount of herbicide leached, as shown by reduced shoot length of the bioassay species. There were no differences between rainfall intensities. Multiple rainfall events (4 × 5 mm over two days) leached slightly less of the intercepted herbicide from the wheat residue than a single event of 20 mm.

Rainfall was effective at leaching pyroxasulfone from the residue into the soil, even in heavy residues (4 t ha^–1^) when rainfall occurred up to 14 days after herbicide application. Rainfall leached less prosulfocarb, and this only occurred with rain up to 7 days after application of the chemical. Trifluralin leached the least or was lost from the residue, with little improvement in ‘weed’ control when rainfall occurred one day after herbicide application.

## Supporting information

S1 Table Raw data residue and soil used in the Figs 1–7 (Experiment1). (XLSX)

S2 Table Raw data residue and soil used in the Fig 8 (Experiment2). (XLSX)

S1 Fig Original figures of Figs 1–7 cited in the text (Experiment_1). (XLSX)

S2 Fig Original figure of Fig 8 of cited in the text (Experiment_2). (XLSX)

S1 Text Analyses output of data from Experiment_1. (DOCX)

S2 Text Analyses output of data from Experiment_2. (DOCX)
