# Supplementary material for: Rainfall affects leaching of pre-emergent herbicide from wheat residue into the soil
Source: PLoS One. 2019 Feb 1;14(2):e0210219. doi: 10.1371/journal.pone.0210219 (PMC6358059; doi:10.1371/journal.pone.0210219)
Supplement: S1 Text — (DOCX) [file pone.0210219.s005.docx]

Analysis of variance (Pro-Res-AR)

Variate: SI%_of_Cntrol

Source of variation d.f. s.s. m.s. v.r. F pr.

rep stratum 3 462.1 154.0 0.81

rep.PlotNo stratum

R_Int 2 2567.4 1283.7 6.77 0.001

R_Amount 3 3313.7 1104.6 5.82 <.001

A_Time 4 21352.0 5338.0 28.15 <.001

R_Int.R_Amount 6 2018.5 336.4 1.77 0.107

R_Int.A_Time 8 2297.3 287.2 1.51 0.155

R_Amount.A_Time 12 5664.5 472.0 2.49 0.005

R_Int.R_Amount.A_Time 24 3063.5 127.6 0.67 0.873

Residual 177 33564.1 189.6

Total 239 74303.0

Tables of means

Variate: SI%_of_Cntrol

Grand mean 7.19

R_Int 5 10 20

6.27 3.73 11.58

R_Amount 0 10 20 5 UTC

1.83 9.19 11.77 5.98

A_Time 0.00 0.25 1.00 7.00 14.00

0.00 0.44 1.79 8.77 24.96

R_Int R_Amount 0 10 20 5 UTC

5 0.58 8.87 10.14 5.50

10 0.57 7.68 2.57 4.10

20 4.33 11.03 22.60 8.35

R_Int A_Time 0.00 0.25 1.00 7.00 14.00

5 0.00 0.50 0.00 8.59 22.26

10 0.00 0.00 0.00 0.73 17.91

20 0.00 0.82 5.36 17.00 34.71

R_Amount A_Time 0.00 0.25 1.00 7.00 14.00

0 0.00 0.00 0.00 0.97 8.16

10 0.00 0.00 0.00 7.55 38.42

20 0.00 0.67 7.15 17.00 34.03

5 0.00 1.09 0.00 9.58 19.24

R_Int R_Amount A_Time 0.00 0.25 1.00 7.00 14.00

5 0 0.00 0.00 0.00 2.91 0.00

10 0.00 0.00 0.00 9.83 34.52

20 0.00 2.01 0.00 12.90 35.79

5 0.00 0.00 0.00 8.72 18.75

10 0 0.00 0.00 0.00 0.00 2.83

10 0.00 0.00 0.00 2.91 35.51

20 0.00 0.00 0.00 0.00 12.84

5 0.00 0.00 0.00 0.00 20.49

20 0 0.00 0.00 0.00 0.00 21.66

10 0.00 0.00 0.00 9.92 45.23

20 0.00 0.00 21.46 38.09 53.47

5 0.00 3.26 0.00 20.01 18.48

Standard errors of differences of means

Table R_Int R_Amount A_Time R_Int

R_Amount

rep. 80 60 48 20

d.f. 177 177 177 177

s.e.d. 2.177 2.514 2.811 4.355

Table R_Int R_Amount R_Int

A_Time A_Time R_Amount

A_Time

rep. 16 12 4

d.f. 177 177 177

s.e.d. 4.869 5.622 9.737

Least significant differences of means (5% level)

Table R_Int R_Amount A_Time R_Int

R_Amount

rep. 80 60 48 20

d.f. 177 177 177 177

l.s.d. 4.297 4.962 5.547 8.594

Table R_Int R_Amount R_Int

A_Time A_Time R_Amount

A_Time

rep. 16 12 4

d.f. 177 177 177

l.s.d. 9.608 11.094 19.216

Stratum standard errors and coefficients of variation

Variate: SI%_of_Cntrol

Stratum d.f. s.e. cv%

rep 3 1.602 22.3

rep.PlotNo 177 13.771 191.4

Analysis of variance (Pro-Soil-AR)

Variate: SI%_of_Cntrol

Source of variation d.f. s.s. m.s. v.r. F pr.

rep stratum 3 599.6 199.9 0.84

rep.PlotNo stratum

R_Int 2 2302.1 1151.0 4.82 0.009

R_Amount 3 22335.4 7445.1 31.17 <.001

A_Time 4 16952.4 4238.1 17.74 <.001

R_Int.R_Amount 6 2805.1 467.5 1.96 0.074

R_Int.A_Time 8 18440.2 2305.0 9.65 <.001

R_Amount.A_Time 12 11880.8 990.1 4.15 <.001

R_Int.R_Amount.A_Time 24 5773.3 240.6 1.01 0.460

Residual 177 42277.5 238.9

Total 239 123366.4

Tables of means

Variate: SI%_of_Cntrol

Grand mean 44.

R_Int 5 10 20

45. 46. 39.

R_Amount 0 10 20 5 UTC

60. 37. 37. 41.

A_Time 0.00 0.25 1.00 7.00 14.00

34. 41. 41. 42. 59.

R_Int R_Amount 0 10 20 5 UTC

5 55. 38. 45. 43.

10 65. 38. 36. 46.

20 60. 34. 30. 33.

R_Int A_Time 0.00 0.25 1.00 7.00 14.00

5 33. 33. 41. 46. 74.

10 50. 55. 46. 31. 50.

20 20. 37. 36. 49. 54.

R_Amount A_Time 0.00 0.25 1.00 7.00 14.00

0 69. 58. 57. 57. 59.

10 26. 38. 30. 31. 59.

20 19. 37. 33. 36. 60.

5 23. 33. 42. 45. 60.

R_Int R_Amount A_Time 0.00 0.25 1.00 7.00 14.00

5 0 64. 33. 52. 59. 69.

10 21. 31. 31. 31. 77.

20 21. 39. 41. 40. 81.

5 25. 26. 39. 55. 71.

10 0 92. 72. 61. 42. 57.

10 40. 53. 29. 28. 40.

20 37. 44. 37. 19. 44.

5 30. 48. 57. 36. 59.

20 0 50. 68. 59. 72. 51.

10 17. 30. 31. 32. 60.

20 0. 26. 22. 48. 54.

5 14. 24. 32. 44. 51.

Standard errors of differences of means

Table R_Int R_Amount A_Time R_Int

R_Amount

rep. 80 60 48 20

d.f. 177 177 177 177

s.e.d. 2.4 2.8 3.2 4.9

Table R_Int R_Amount R_Int

A_Time A_Time R_Amount

A_Time

rep. 16 12 4

d.f. 177 177 177

s.e.d. 5.5 6.3 10.9

Least significant differences of means (5% level)

Table R_Int R_Amount A_Time R_Int

R_Amount

rep. 80 60 48 20

d.f. 177 177 177 177

l.s.d. 4.8 5.6 6.2 9.6

Table R_Int R_Amount R_Int

A_Time A_Time R_Amount

A_Time

rep. 16 12 4

d.f. 177 177 177

l.s.d. 10.8 12.5 21.6

Stratum standard errors and coefficients of variation

Variate: SI%_of_Cntrol

Stratum d.f. s.e. cv%

rep 3 1.8 4.2

rep.PlotNo 177 15.5 35.4

Analysis of variance (Pro-Res-CU)

Variate: SI%_of_Cntrol

Source of variation d.f. s.s. m.s. v.r. F pr.

rep stratum 3 381.7 127.2 0.86

rep.PlotNo stratum

R_Int 2 76.4 38.2 0.26 0.773

R_Amount 3 5774.2 1924.7 13.03 <.001

A_Time 4 23739.0 5934.8 40.16 <.001

R_Int.R_Amount 6 560.8 93.5 0.63 0.704

R_Int.A_Time 8 1470.3 183.8 1.24 0.276

R_Amount.A_Time 12 2200.5 183.4 1.24 0.258

R_Int.R_Amount.A_Time 24 2108.3 87.8 0.59 0.933

Residual 177 26153.6 147.8

Total 239 62464.8

Tables of means

Variate: SI%_of_Cntrol

Grand mean 60.17

R_Int 5 10 20

59.39 60.40 60.72

R_Amount 0 10 20 5 UTC

55.64 61.53 67.62 55.88

A_Time 0.00 0.25 1.00 7.00 14.00

48.88 49.44 60.51 68.10 73.92

R_Int R_Amount 0 10 20 5 UTC

5 54.68 61.74 65.66 55.50

10 54.97 63.49 66.18 56.95

20 57.28 59.36 71.02 55.20

R_Int A_Time 0.00 0.25 1.00 7.00 14.00

5 44.56 49.76 62.12 68.47 72.05

10 46.47 49.32 59.43 69.29 77.47

20 55.59 49.25 59.98 66.53 72.24

R_Amount A_Time 0.00 0.25 1.00 7.00 14.00

0 43.17 50.31 54.39 66.45 63.89

10 54.64 48.45 62.69 68.35 73.52

20 57.87 53.30 70.15 73.59 83.20

5 39.83 45.72 54.80 64.01 75.06

R_Int R_Amount A_Time 0.00 0.25 1.00 7.00 14.00

5 0 44.28 48.91 57.50 61.97 60.74

10 48.42 54.55 63.24 69.03 73.43

20 51.27 52.65 70.37 74.48 79.53

5 34.28 42.92 57.38 68.41 74.49

10 0 40.57 48.54 51.75 64.32 69.65

10 50.44 47.89 61.60 75.09 82.43

20 53.75 52.17 68.23 76.33 80.43

5 41.13 48.67 56.16 61.41 77.36

20 0 44.66 53.49 53.94 73.06 61.27

10 65.06 42.90 63.24 60.92 64.70

20 68.60 55.06 71.85 69.94 89.64

5 44.06 45.57 50.87 62.20 73.32

Standard errors of differences of means

Table R_Int R_Amount A_Time R_Int

R_Amount

rep. 80 60 48 20

d.f. 177 177 177 177

s.e.d. 1.922 2.219 2.481 3.844

Table R_Int R_Amount R_Int

A_Time A_Time R_Amount

A_Time

rep. 16 12 4

d.f. 177 177 177

s.e.d. 4.298 4.963 8.595

Least significant differences of means (5% level)

Table R_Int R_Amount A_Time R_Int

R_Amount

rep. 80 60 48 20

d.f. 177 177 177 177

l.s.d. 3.793 4.380 4.897 7.586

Table R_Int R_Amount R_Int

A_Time A_Time R_Amount

A_Time

rep. 16 12 4

d.f. 177 177 177

l.s.d. 8.481 9.793 16.963

Stratum standard errors and coefficients of variation

Variate: SI%_of_Cntrol

Stratum d.f. s.e. cv%

rep 3 1.456 2.4

rep.PlotNo 177 12.156 20.2

Analysis of variance (Pro-Soil-CU)

Variate: SI%_of_Cntrol

Source of variation d.f. s.s. m.s. v.r. F pr.

rep stratum 3 330.9 110.3 0.91

rep.PlotNo stratum

R_Int 2 1249.8 624.9 5.14 0.007

R_Amount 3 2824.0 941.3 7.75 <.001

A_Time 4 2233.9 558.5 4.60 0.001

R_Int.R_Amount 6 440.3 73.4 0.60 0.727

R_Int.A_Time 8 3816.8 477.1 3.93 <.001

R_Amount.A_Time 12 5125.7 427.1 3.52 <.001

R_Int.R_Amount.A_Time 24 6196.2 258.2 2.13 0.003

Residual 177 21501.0 121.5

Total 239 43718.6

Tables of means

Variate: SI%_of_Cntrol

Grand mean 62.

R_Int 5 10 20

62. 65. 59.

R_Amount 0 10 20 5 UTC

67. 60. 58. 63.

A_Time 0.00 0.25 1.00 7.00 14.00

56. 64. 61. 63. 64.

R_Int R_Amount 0 10 20 5 UTC

5 65. 60. 57. 65.

10 71. 64. 59. 65.

20 63. 56. 57. 60.

R_Int A_Time 0.00 0.25 1.00 7.00 14.00

5 59. 55. 60. 64. 70.

10 59. 76. 63. 63. 62.

20 52. 62. 59. 62. 61.

R_Amount A_Time 0.00 0.25 1.00 7.00 14.00

0 75. 69. 65. 62. 63.

10 47. 62. 62. 64. 65.

20 49. 60. 57. 57. 64.

5 55. 68. 58. 70. 66.

R_Int R_Amount A_Time 0.00 0.25 1.00 7.00 14.00

5 0 81. 61. 58. 56. 71.

10 41. 53. 62. 67. 74.

20 43. 43. 64. 60. 72.

5 70. 64. 56. 74. 63.

10 0 81. 80. 73. 59. 61.

10 57. 75. 67. 64. 58.

20 48. 77. 57. 52. 60.

5 48. 73. 56. 77. 70.

20 0 62. 65. 65. 70. 56.

10 43. 57. 56. 59. 63.

20 56. 59. 50. 60. 60.

5 46. 66. 64. 59. 65.

Standard errors of differences of means

Table R_Int R_Amount A_Time R_Int

R_Amount

rep. 80 60 48 20

d.f. 177 177 177 177

s.e.d. 1.7 2.0 2.2 3.5

Table R_Int R_Amount R_Int

A_Time A_Time R_Amount

A_Time

rep. 16 12 4

d.f. 177 177 177

s.e.d. 3.9 4.5 7.8

Least significant differences of means (5% level)

Table R_Int R_Amount A_Time R_Int

R_Amount

rep. 80 60 48 20

d.f. 177 177 177 177

l.s.d. 3.4 4.0 4.4 6.9

Table R_Int R_Amount R_Int

A_Time A_Time R_Amount

A_Time

rep. 16 12 4

d.f. 177 177 177

l.s.d. 7.7 8.9 15.4

Stratum standard errors and coefficients of variation

Variate: SI%_of_Cntrol

Stratum d.f. s.e. cv%

rep 3 1.4 2.2

rep.PlotNo 177 11.0 17.8

Analysis of variance (Pyro-Res-AR)

Variate: SI%_of_Cntrol

Source of variation d.f. s.s. m.s. v.r. F pr.

rep stratum 3 4.3815 1.4605 1.98

rep.PlotNo stratum

R_Int 2 0.7852 0.3926 0.53 0.588

R_Amount 3 1.5337 0.5112 0.69 0.557

A_Time 4 2.2823 0.5706 0.77 0.543

R_Int.R_Amount 6 5.2033 0.8672 1.18 0.320

R_Int.A_Time 8 6.7004 0.8376 1.14 0.341

R_Amount.A_Time 12 9.6946 0.8079 1.10 0.365

R_Int.R_Amount.A_Time 24 17.2535 0.7189 0.98 0.500

Residual 177 130.3590 0.7365

Total 239 178.1935

Tables of means

Variate: SI%_of_Cntrol

Grand mean 0.08

R_Int 5 10 20

0.00 0.10 0.14

R_Amount 0 10 20 5 UTC

0.00 0.13 0.00 0.18

A_Time 0.00 0.25 1.00 7.00 14.00

0.00 0.16 0.23 0.00 0.00

R_Int R_Amount 0 10 20 5 UTC

5 0.00 0.00 0.00 0.00

10 0.00 0.39 0.00 0.00

20 0.00 0.00 0.00 0.54

R_Int A_Time 0.00 0.25 1.00 7.00 14.00

5 0.00 0.00 0.00 0.00 0.00

10 0.00 0.49 0.00 0.00 0.00

20 0.00 0.00 0.68 0.00 0.00

R_Amount A_Time 0.00 0.25 1.00 7.00 14.00

0 0.00 0.00 0.00 0.00 0.00

10 0.00 0.66 0.00 0.00 0.00

20 0.00 0.00 0.00 0.00 0.00

5 0.00 0.00 0.90 0.00 0.00

R_Int R_Amount A_Time 0.00 0.25 1.00 7.00 14.00

5 0 0.00 0.00 0.00 0.00 0.00

10 0.00 0.00 0.00 0.00 0.00

20 0.00 0.00 0.00 0.00 0.00

5 0.00 0.00 0.00 0.00 0.00

10 0 0.00 0.00 0.00 0.00 0.00

10 0.00 1.97 0.00 0.00 0.00

20 0.00 0.00 0.00 0.00 0.00

5 0.00 0.00 0.00 0.00 0.00

20 0 0.00 0.00 0.00 0.00 0.00

10 0.00 0.00 0.00 0.00 0.00

20 0.00 0.00 0.00 0.00 0.00

5 0.00 0.00 2.71 0.00 0.00

Standard errors of differences of means

Table R_Int R_Amount A_Time R_Int

R_Amount

rep. 80 60 48 20

d.f. 177 177 177 177

s.e.d. 0.136 0.157 0.175 0.271

Table R_Int R_Amount R_Int

A_Time A_Time R_Amount

A_Time

rep. 16 12 4

d.f. 177 177 177

s.e.d. 0.303 0.350 0.607

Least significant differences of means (5% level)

Table R_Int R_Amount A_Time R_Int

R_Amount

rep. 80 60 48 20

d.f. 177 177 177 177

l.s.d. 0.268 0.309 0.346 0.536

Table R_Int R_Amount R_Int

A_Time A_Time R_Amount

A_Time

rep. 16 12 4

d.f. 177 177 177

l.s.d. 0.599 0.691 1.198

Stratum standard errors and coefficients of variation

Variate: SI%_of_Cntrol

Stratum d.f. s.e. cv%

rep 3 0.156 200.0

rep.PlotNo 177 0.858 1100.1

Analysis of variance (Pyro-Soil-AR)

Variate: SI%_of_Cntrol

Source of variation d.f. s.s. m.s. v.r. F pr.

rep stratum 3 6.73 2.24 0.15

rep.PlotNo stratum

R_Int 2 1016.04 508.02 35.06 <.001

R_Amount 3 85507.12 28502.37 1966.92 <.001

A_Time 4 5555.11 1388.78 95.84 <.001

R_Int.R_Amount 6 3087.12 514.52 35.51 <.001

R_Int.A_Time 8 855.38 106.92 7.38 <.001

R_Amount.A_Time 12 17365.57 1447.13 99.87 <.001

R_Int.R_Amount.A_Time 24 2216.03 92.33 6.37 <.001

Residual 177 2564.88 14.49

Total 239 118173.97

Tables of means

Variate: SI%_of_Cntrol

Grand mean 11.

R_Int 5 10 20

10. 9. 14.

R_Amount 0 10 20 5 UTC

44. 0. 0. 1.

A_Time 0.00 0.25 1.00 7.00 14.00

5. 8. 11. 13. 19.

R_Int R_Amount 0 10 20 5 UTC

5 39. 0. 0. 1.

10 38. 0. 0. 0.

20 55. 0. 0. 1.

R_Int A_Time 0.00 0.25 1.00 7.00 14.00

5 4. 7. 13. 12. 14.

10 4. 6. 9. 9. 19.

20 7. 12. 10. 18. 24.

R_Amount A_Time 0.00 0.25 1.00 7.00 14.00

0 18. 33. 41. 51. 77.

10 0. 0. 0. 0. 0.

20 0. 0. 0. 1. 0.

5 1. 0. 2. 0. 0.

R_Int R_Amount A_Time 0.00 0.25 1.00 7.00 14.00

5 0 15. 29. 46. 46. 57.

10 0. 0. 0. 0. 0.

20 0. 0. 0. 2. 0.

5 0. 0. 6. 0. 0.

10 0 16. 24. 35. 36. 77.

10 0. 0. 0. 0. 0.

20 0. 0. 0. 0. 0.

5 0. 0. 0. 0. 0.

20 0 24. 47. 41. 71. 95.

10 0. 0. 0. 0. 0.

20 0. 0. 0. 0. 0.

5 3. 0. 0. 0. 0.

Standard errors of differences of means

Table R_Int R_Amount A_Time R_Int

R_Amount

rep. 80 60 48 20

d.f. 177 177 177 177

s.e.d. 0.6 0.7 0.8 1.2

Table R_Int R_Amount R_Int

A_Time A_Time R_Amount

A_Time

rep. 16 12 4

d.f. 177 177 177

s.e.d. 1.3 1.6 2.7

Least significant differences of means (5% level)

Table R_Int R_Amount A_Time R_Int

R_Amount

rep. 80 60 48 20

d.f. 177 177 177 177

l.s.d. 1.2 1.4 1.5 2.4

Table R_Int R_Amount R_Int

A_Time A_Time R_Amount

A_Time

rep. 16 12 4

d.f. 177 177 177

l.s.d. 2.7 3.1 5.3

Stratum standard errors and coefficients of variation

Variate: SI%_of_Cntrol

Stratum d.f. s.e. cv%

rep 3 0.2 1.7

rep.PlotNo 177 3.8 34.1

Analysis of variance (Pyro-Res-CU)

Variate: SI%_of_Cntrol

Source of variation d.f. s.s. m.s. v.r. F pr.

rep stratum 3 241.99 80.66 0.85

rep.PlotNo stratum

R_Int 2 745.02 372.51 3.91 0.022

R_Amount 3 5825.32 1941.77 20.40 <.001

A_Time 4 6855.47 1713.87 18.00 <.001

R_Int.R_Amount 6 941.31 156.89 1.65 0.137

R_Int.A_Time 8 2121.39 265.17 2.79 0.006

R_Amount.A_Time 12 4315.64 359.64 3.78 <.001

R_Int.R_Amount.A_Time 24 3840.13 160.01 1.68 0.031

Residual 177 16849.51 95.19

Total 239 41735.79

Tables of means

Variate: SI%_of_Cntrol

Grand mean 57.27

R_Int 5 10 20

54.84 58.01 58.96

R_Amount 0 10 20 5 UTC

50.60 59.89 63.63 54.96

A_Time 0.00 0.25 1.00 7.00 14.00

49.71 55.07 55.25 61.23 65.09

R_Int R_Amount 0 10 20 5 UTC

5 47.72 59.92 60.65 51.07

10 50.35 62.16 62.02 57.50

20 53.72 57.60 68.20 56.32

R_Int A_Time 0.00 0.25 1.00 7.00 14.00

5 51.49 53.97 52.33 60.08 56.32

10 49.98 56.63 53.57 63.10 66.76

20 47.65 54.62 59.86 60.50 72.17

R_Amount A_Time 0.00 0.25 1.00 7.00 14.00

0 42.63 52.03 37.04 54.92 66.38

10 53.34 59.26 62.95 60.18 63.72

20 53.83 60.81 67.86 67.91 67.72

5 49.03 48.20 53.16 61.90 62.52

R_Int R_Amount A_Time 0.00 0.25 1.00 7.00 14.00

5 0 45.97 53.18 30.49 54.22 54.75

10 59.85 57.50 63.02 60.83 58.38

20 54.39 58.18 66.31 69.17 55.22

5 45.76 47.04 49.50 56.09 56.95

10 0 39.12 52.01 34.91 53.00 72.71

10 54.67 71.86 61.60 59.72 62.94

20 52.94 56.32 69.73 70.99 60.11

5 53.21 46.33 48.01 68.68 71.30

20 0 42.82 50.89 45.70 57.52 71.68

10 45.50 48.42 64.22 60.00 69.86

20 54.15 67.93 67.55 63.56 87.82

5 48.12 51.24 61.98 60.93 59.33

Standard errors of differences of means

Table R_Int R_Amount A_Time R_Int

R_Amount

rep. 80 60 48 20

d.f. 177 177 177 177

s.e.d. 1.543 1.781 1.992 3.085

Table R_Int R_Amount R_Int

A_Time A_Time R_Amount

A_Time

rep. 16 12 4

d.f. 177 177 177

s.e.d. 3.450 3.983 6.899

Least significant differences of means (5% level)

Table R_Int R_Amount A_Time R_Int

R_Amount

rep. 80 60 48 20

d.f. 177 177 177 177

l.s.d. 3.044 3.515 3.930 6.089

Table R_Int R_Amount R_Int

A_Time A_Time R_Amount

A_Time

rep. 16 12 4

d.f. 177 177 177

l.s.d. 6.808 7.861 13.615

Stratum standard errors and coefficients of variation

Variate: SI%_of_Cntrol

Stratum d.f. s.e. cv%

rep 3 1.159 2.0

rep.PlotNo 177 9.757 17.0

Analysis of variance (Pyro-Soil-CU)

Variate: SI%_of_Cntrol

Source of variation d.f. s.s. m.s. v.r. F pr.

rep stratum 3 238.6 79.5 0.64

rep.PlotNo stratum

R_Int 2 363.6 181.8 1.46 0.236

R_Amount 3 23363.3 7787.8 62.44 <.001

A_Time 4 28253.0 7063.3 56.63 <.001

R_Int.R_Amount 6 711.3 118.5 0.95 0.461

R_Int.A_Time 8 6652.6 831.6 6.67 <.001

R_Amount.A_Time 12 1295.6 108.0 0.87 0.583

R_Int.R_Amount.A_Time 24 4473.9 186.4 1.49 0.074

Residual 177 22077.4 124.7

Total 239 87429.3

Tables of means

Variate: SI%_of_Cntrol

Grand mean 42.

R_Int 5 10 20

42. 40. 43.

R_Amount 0 10 20 5 UTC

58. 34. 35. 39.

A_Time 0.00 0.25 1.00 7.00 14.00

26. 34. 42. 54. 53.

R_Int R_Amount 0 10 20 5 UTC

5 60. 34. 31. 42.

10 57. 33. 34. 36.

20 58. 36. 39. 38.

R_Int A_Time 0.00 0.25 1.00 7.00 14.00

5 19. 35. 46. 51. 57.

10 26. 33. 40. 45. 55.

20 32. 33. 39. 66. 45.

R_Amount A_Time 0.00 0.25 1.00 7.00 14.00

0 35. 52. 62. 71. 72.

10 20. 26. 33. 47. 45.

20 20. 27. 34. 47. 46.

5 28. 29. 38. 52. 47.

R_Int R_Amount A_Time 0.00 0.25 1.00 7.00 14.00

5 0 26. 52. 67. 71. 85.

10 19. 30. 37. 42. 41.

20 13. 29. 32. 32. 50.

5 17. 31. 48. 60. 51.

10 0 35. 49. 60. 58. 81.

10 18. 24. 32. 41. 49.

20 20. 26. 36. 44. 44.

5 32. 33. 33. 36. 48.

20 0 45. 56. 57. 85. 49.

10 22. 25. 31. 56. 46.

20 27. 25. 35. 64. 43.

5 34. 24. 32. 59. 43.

Standard errors of differences of means

Table R_Int R_Amount A_Time R_Int

R_Amount

rep. 80 60 48 20

d.f. 177 177 177 177

s.e.d. 1.8 2.0 2.3 3.5

Table R_Int R_Amount R_Int

A_Time A_Time R_Amount

A_Time

rep. 16 12 4

d.f. 177 177 177

s.e.d. 3.9 4.6 7.9

Least significant differences of means (5% level)

Table R_Int R_Amount A_Time R_Int

R_Amount

rep. 80 60 48 20

d.f. 177 177 177 177

l.s.d. 3.5 4.0 4.5 7.0

Table R_Int R_Amount R_Int

A_Time A_Time R_Amount

A_Time

rep. 16 12 4

d.f. 177 177 177

l.s.d. 7.8 9.0 15.6

Stratum standard errors and coefficients of variation

Variate: SI%_of_Cntrol

Stratum d.f. s.e. cv%

rep 3 1.2 2.8

rep.PlotNo 177 11.2 26.8

Analysis of variance (Tri-Res-AR)

Variate: SI%_of_Cntrol

Source of variation d.f. s.s. m.s. v.r. F pr.

rep stratum 3 2009.55 669.85 7.86

rep.PlotNo stratum

R_Int 2 329.01 164.50 1.93 0.148

R_Amount 3 726.89 242.30 2.84 0.039

A_Time 4 249927.00 62481.75 732.79 <.001

R_Int.R_Amount 6 558.18 93.03 1.09 0.370

R_Int.A_Time 8 1340.91 167.61 1.97 0.053

R_Amount.A_Time 12 1148.52 95.71 1.12 0.345

R_Int.R_Amount.A_Time 24 1607.82 66.99 0.79 0.751

Residual 177 15092.08 85.27

Total 239 272739.96

Tables of means

Variate: SI%_of_Cntrol

Grand mean 50.29

R_Int 5 10 20

51.57 48.74 50.56

R_Amount 0 10 20 5 UTC

48.84 49.17 53.23 49.94

A_Time 0.00 0.25 1.00 7.00 14.00

4.76 34.03 38.49 83.93 90.26

R_Int R_Amount 0 10 20 5 UTC

5 51.72 50.61 52.79 51.17

10 46.39 45.43 52.42 50.73

20 48.40 51.46 54.46 47.92

R_Int A_Time 0.00 0.25 1.00 7.00 14.00

5 5.24 33.82 40.66 85.22 92.93

10 7.71 34.95 32.54 81.97 86.55

20 1.33 33.31 42.26 84.61 91.30

R_Amount A_Time 0.00 0.25 1.00 7.00 14.00

0 0.00 34.79 37.30 84.22 87.87

10 4.25 29.70 40.82 81.16 89.92

20 8.33 37.25 42.95 84.11 93.50

5 6.45 34.36 32.88 86.25 89.77

R_Int R_Amount A_Time 0.00 0.25 1.00 7.00 14.00

5 0 0.00 37.30 46.88 85.99 88.43

10 10.05 26.81 41.15 81.90 93.13

20 3.16 36.50 43.11 84.08 97.13

5 7.75 34.66 31.49 88.93 93.03

10 0 0.00 33.70 30.75 79.13 88.36

10 2.71 26.93 33.44 79.66 84.44

20 16.52 39.99 33.36 84.23 88.02

5 11.60 39.19 32.62 84.84 85.40

20 0 0.00 33.38 34.25 87.55 86.81

10 0.00 35.35 47.87 81.92 92.19

20 5.30 35.26 52.39 84.01 95.34

5 0.00 29.24 34.53 84.98 90.86

Standard errors of differences of means

Table R_Int R_Amount A_Time R_Int

R_Amount

rep. 80 60 48 20

d.f. 177 177 177 177

s.e.d. 1.460 1.686 1.885 2.920

Table R_Int R_Amount R_Int

A_Time A_Time R_Amount

A_Time

rep. 16 12 4

d.f. 177 177 177

s.e.d. 3.265 3.770 6.529

Least significant differences of means (5% level)

Table R_Int R_Amount A_Time R_Int

R_Amount

rep. 80 60 48 20

d.f. 177 177 177 177

l.s.d. 2.881 3.327 3.720 5.763

Table R_Int R_Amount R_Int

A_Time A_Time R_Amount

A_Time

rep. 16 12 4

d.f. 177 177 177

l.s.d. 6.443 7.439 12.885

Stratum standard errors and coefficients of variation

Variate: SI%_of_Cntrol

Stratum d.f. s.e. cv%

rep 3 3.341 6.6

rep.PlotNo 177 9.234 18.4

Analysis of variance (Tri-Soil-AR)

Variate: SI%_of_Cntrol

Source of variation d.f. s.s. m.s. v.r. F pr.

rep stratum 3 781.9 260.6 0.81

rep.PlotNo stratum

R_Int 2 2056.4 1028.2 3.18 0.044

R_Amount 3 5654.2 1884.7 5.83 <.001

A_Time 4 4358.9 1089.7 3.37 0.011

R_Int.R_Amount 6 2236.4 372.7 1.15 0.334

R_Int.A_Time 8 13121.9 1640.2 5.07 <.001

R_Amount.A_Time 12 8102.1 675.2 2.09 0.020

R_Int.R_Amount.A_Time 24 6072.5 253.0 0.78 0.756

Residual 177 57240.2 323.4

Total 239 99624.5

Tables of means

Variate: SI%_of_Cntrol

Grand mean 44.

R_Int 5 10 20

46. 46. 40.

R_Amount 0 10 20 5 UTC

51. 43. 44. 37.

A_Time 0.00 0.25 1.00 7.00 14.00

36. 46. 43. 44. 49.

R_Int R_Amount 0 10 20 5 UTC

5 50. 49. 45. 37.

10 59. 41. 47. 37.

20 42. 39. 41. 36.

R_Int A_Time 0.00 0.25 1.00 7.00 14.00

5 38. 32. 51. 49. 58.

10 40. 61. 49. 39. 41.

20 30. 43. 30. 45. 50.

R_Amount A_Time 0.00 0.25 1.00 7.00 14.00

0 53. 58. 49. 49. 44.

10 30. 38. 46. 45. 58.

20 29. 52. 39. 47. 56.

5 34. 35. 39. 37. 40.

R_Int R_Amount A_Time 0.00 0.25 1.00 7.00 14.00

5 0 63. 38. 60. 44. 46.

10 29. 36. 59. 58. 65.

20 29. 32. 44. 57. 63.

5 33. 21. 38. 38. 57.

10 0 65. 86. 52. 48. 44.

10 25. 48. 48. 32. 54.

20 36. 65. 49. 41. 43.

5 36. 46. 46. 35. 25.

20 0 31. 50. 35. 54. 42.

10 35. 29. 30. 44. 56.

20 23. 57. 22. 43. 61.

5 33. 37. 32. 38. 39.

Standard errors of differences of means

Table R_Int R_Amount A_Time R_Int

R_Amount

rep. 80 60 48 20

d.f. 177 177 177 177

s.e.d. 2.8 3.3 3.7 5.7

Table R_Int R_Amount R_Int

A_Time A_Time R_Amount

A_Time

rep. 16 12 4

d.f. 177 177 177

s.e.d. 6.4 7.3 12.7

Least significant differences of means (5% level)

Table R_Int R_Amount A_Time R_Int

R_Amount

rep. 80 60 48 20

d.f. 177 177 177 177

l.s.d. 5.6 6.5 7.2 11.2

Table R_Int R_Amount R_Int

A_Time A_Time R_Amount

A_Time

rep. 16 12 4

d.f. 177 177 177

l.s.d. 12.5 14.5 25.1

Stratum standard errors and coefficients of variation

Variate: SI%_of_Cntrol

Stratum d.f. s.e. cv%

rep 3 2.1 4.8

rep.PlotNo 177 18.0 41.1

Analysis of variance (Tri-Res-CU)

Variate: SI%_of_Cntrol

Source of variation d.f. s.s. m.s. v.r. F pr.

rep stratum 3 834.5 278.2 2.54

rep.PlotNo stratum

R_Int 2 1602.0 801.0 7.31 <.001

R_Amount 3 5300.6 1766.9 16.12 <.001

A_Time 4 37009.4 9252.4 84.42 <.001

R_Int.R_Amount 6 486.4 81.1 0.74 0.618

R_Int.A_Time 8 2219.1 277.4 2.53 0.012

R_Amount.A_Time 12 3190.4 265.9 2.43 0.006

R_Int.R_Amount.A_Time 24 3656.1 152.3 1.39 0.117

Residual 177 19399.0 109.6

Total 239 73697.5

Tables of means

Variate: SI%_of_Cntrol

Grand mean 70.41

R_Int 5 10 20

66.81 72.74 71.68

R_Amount 0 10 20 5 UTC

64.03 74.24 75.54 67.84

A_Time 0.00 0.25 1.00 7.00 14.00

49.65 64.02 76.83 76.16 85.40

R_Int R_Amount 0 10 20 5 UTC

5 62.95 68.98 73.00 62.31

10 64.32 78.08 76.84 71.73

20 64.81 75.67 76.78 69.47

R_Int A_Time 0.00 0.25 1.00 7.00 14.00

5 49.59 59.01 74.91 76.02 74.51

10 48.60 66.05 78.41 77.39 93.26

20 50.74 66.99 77.16 75.08 88.43

R_Amount A_Time 0.00 0.25 1.00 7.00 14.00

0 38.91 56.94 68.34 75.46 80.48

10 51.83 69.34 82.39 80.59 87.06

20 54.98 67.30 78.70 85.65 91.06

5 52.86 62.49 77.89 62.94 83.00

R_Int R_Amount A_Time 0.00 0.25 1.00 7.00 14.00

5 0 35.21 54.59 69.00 87.43 68.55

10 50.89 68.94 73.88 75.66 75.52

20 56.76 65.34 77.63 83.07 82.20

5 55.52 47.18 79.13 57.92 71.79

10 0 36.50 58.39 70.98 65.54 90.21

10 48.82 72.03 88.35 84.97 96.24

20 59.03 68.79 74.30 87.86 94.20

5 50.05 65.01 80.03 71.17 92.38

20 0 45.03 57.84 65.04 73.42 82.69

10 55.78 67.07 84.95 81.14 89.43

20 49.15 67.77 84.15 86.04 96.77

5 53.00 75.29 74.51 59.73 84.84

Standard errors of differences of means

Table R_Int R_Amount A_Time R_Int

R_Amount

rep. 80 60 48 20

d.f. 177 177 177 177

s.e.d. 1.655 1.911 2.137 3.311

Table R_Int R_Amount R_Int

A_Time A_Time R_Amount

A_Time

rep. 16 12 4

d.f. 177 177 177

s.e.d. 3.701 4.274 7.403

Least significant differences of means (5% level)

Table R_Int R_Amount A_Time R_Int

R_Amount

rep. 80 60 48 20

d.f. 177 177 177 177

l.s.d. 3.267 3.772 4.217 6.533

Table R_Int R_Amount R_Int

A_Time A_Time R_Amount

A_Time

rep. 16 12 4

d.f. 177 177 177

l.s.d. 7.304 8.434 14.609

Stratum standard errors and coefficients of variation

Variate: SI%_of_Cntrol

Stratum d.f. s.e. cv%

rep 3 2.153 3.1

rep.PlotNo 177 10.469 14.9

Analysis of variance (Tri-Soil-CU)

Variate: SI%_of_Cntrol

Source of variation d.f. s.s. m.s. v.r. F pr.

rep stratum 3 555.1 185.0 0.76

rep.PlotNo stratum

R_Int 2 999.4 499.7 2.05 0.131

R_Amount 3 2851.2 950.4 3.91 0.010

A_Time 4 1647.5 411.9 1.69 0.153

R_Int.R_Amount 6 4017.9 669.6 2.75 0.014

R_Int.A_Time 8 3055.2 381.9 1.57 0.137

R_Amount.A_Time 12 2743.1 228.6 0.94 0.508

R_Int.R_Amount.A_Time 24 7800.2 325.0 1.34 0.146

Residual 177 43038.9 243.2

Total 239 66708.3

Tables of means

Variate: SI%_of_Cntrol

Grand mean 62.

R_Int 5 10 20

62. 64. 59.

R_Amount 0 10 20 5 UTC

66. 63. 57. 60.

A_Time 0.00 0.25 1.00 7.00 14.00

60. 58. 63. 62. 65.

R_Int R_Amount 0 10 20 5 UTC

5 69. 65. 52. 61.

10 69. 64. 68. 57.

20 61. 61. 51. 64.

R_Int A_Time 0.00 0.25 1.00 7.00 14.00

5 59. 58. 60. 66. 66.

10 66. 54. 72. 61. 67.

20 54. 62. 58. 60. 62.

R_Amount A_Time 0.00 0.25 1.00 7.00 14.00

0 65. 63. 68. 68. 67.

10 56. 60. 71. 60. 69.

20 52. 52. 59. 62. 61.

5 66. 57. 55. 60. 65.

R_Int R_Amount A_Time 0.00 0.25 1.00 7.00 14.00

5 0 70. 69. 67. 76. 62.

10 49. 73. 72. 57. 72.

20 43. 39. 44. 74. 62.

5 72. 50. 59. 57. 69.

10 0 73. 65. 76. 63. 65.

10 61. 42. 85. 62. 67.

20 70. 60. 75. 62. 72.

5 62. 50. 51. 56. 64.

20 0 53. 56. 60. 63. 72.

10 58. 64. 55. 61. 69.

20 42. 56. 59. 49. 48.

5 63. 70. 57. 67. 61.

Standard errors of differences of means

Table R_Int R_Amount A_Time R_Int

R_Amount

rep. 80 60 48 20

d.f. 177 177 177 177

s.e.d. 2.5 2.8 3.2 4.9

Table R_Int R_Amount R_Int

A_Time A_Time R_Amount

A_Time

rep. 16 12 4

d.f. 177 177 177

s.e.d. 5.5 6.4 11.0

Least significant differences of means (5% level)

Table R_Int R_Amount A_Time R_Int

R_Amount

rep. 80 60 48 20

d.f. 177 177 177 177

l.s.d. 4.9 5.6 6.3 9.7

Table R_Int R_Amount R_Int

A_Time A_Time R_Amount

A_Time

rep. 16 12 4

d.f. 177 177 177

l.s.d. 10.9 12.6 21.8

Stratum standard errors and coefficients of variation

Variate: SI%_of_Cntrol

Stratum d.f. s.e. cv%

rep 3 1.8 2.8

rep.PlotNo 177 15.6 25.3
