# Supplementary material for: Rainfall affects leaching of pre-emergent herbicide from wheat residue into the soil
Source: PLoS One. 2019 Feb 1;14(2):e0210219. doi: 10.1371/journal.pone.0210219 (PMC6358059; doi:10.1371/journal.pone.0210219)
Supplement: S2 Text — (DOCX) [file pone.0210219.s006.docx]

Analysis of variance (Pro-Res-AR)

Variate: SL%_of_Ctrl

Source of variation d.f. s.s. m.s. v.r. F pr.

Rep stratum 3 0.00 0.00

Rep.PlotNo stratum

R_Eve 4 32000.00 8000.00

Residual 12 0.00 0.00

Total 19 32000.00

Tables of means

Variate: SL%_of_Ctrl

Grand mean 20.00

R_Eve 0 10+10 20 5+5+5+5

0.00 0.00 0.00 0.00

R_Eve UTC

100.00

Standard errors of differences of means

Table R_Eve

rep. 4

d.f. *

s.e.d. 0.000

Least significant differences of means (5% level)

Table R_Eve

rep. 4

d.f. *

l.s.d. *

Stratum standard errors and coefficients of variation

Variate: SL%_of_Ctrl

Stratum d.f. s.e. cv%

Rep 3 0.000 0.0

Rep.PlotNo 12 0.000 0.0

Analysis of variance (Pro-Res-CU)

Variate: SL%_of_Ctrl

Source of variation d.f. s.s. m.s. v.r. F pr.

Rep stratum 3 618.83 206.28 7.68

Rep.PlotNo stratum

R_Eve 4 5047.04 1261.76 47.00 <.001

Residual 12 322.12 26.84

Total 19 5987.99

Tables of means

Variate: SL%_of_Ctrl

Grand mean 70.3

R_Eve 0 10+10 20 5+5+5+5

54.7 68.7 58.8 69.0

R_Eve UTC

100.0

Standard errors of differences of means

Table R_Eve

rep. 4

d.f. 12

s.e.d. 3.66

Least significant differences of means (5% level)

Table R_Eve

rep. 4

d.f. 12

l.s.d. 7.98

Stratum standard errors and coefficients of variation

Variate: SL%_of_Ctrl

Stratum d.f. s.e. cv%

Rep 3 6.42 9.1

Rep.PlotNo 12 5.18 7.4

Analysis of variance (Pro-Soil-AR)

Variate: SL%_of_Ctrl

Source of variation d.f. s.s. m.s. v.r. F pr.

Rep stratum 3 514.6 171.5 1.16

Rep.PlotNo stratum

R_Eve 4 21052.8 5263.2 35.64 <.001

Residual 12 1772.0 147.7

Total 19 23339.4

Tables of means

Variate: SL%_of_Ctrl

Grand mean 36.66

R_Eve 0 10+10 20 5+5+5+5

33.79 17.22 19.60 12.71

R_Eve UTC

100.00

Standard errors of differences of means

Table R_Eve

rep. 4

d.f. 12

s.e.d. 8.593

Least significant differences of means (5% level)

Table R_Eve

rep. 4

d.f. 12

l.s.d. 18.722

Stratum standard errors and coefficients of variation

Variate: SL%_of_Ctrl

Stratum d.f. s.e. cv%

Rep 3 5.857 16.0

Rep.PlotNo 12 12.152 33.1

Analysis of variance (Pro-Soil-CU)

Variate: SL%_of_Ctrl

Source of variation d.f. s.s. m.s. v.r. F pr.

Rep stratum 3 34.10 11.37 0.35

Rep.PlotNo stratum

R_Eve 4 3913.46 978.36 30.17 <.001

Residual 12 389.09 32.42

Total 19 4336.64

Tables of means

Variate: SL%_of_Ctrl

Grand mean 73.26

R_Eve 0 10+10 20 5+5+5+5

71.32 70.99 62.37 61.61

R_Eve UTC

100.00

Standard errors of differences of means

Table R_Eve

rep. 4

d.f. 12

s.e.d. 4.026

Least significant differences of means (5% level)

Table R_Eve

rep. 4

d.f. 12

l.s.d. 8.773

Stratum standard errors and coefficients of variation

Variate: SL%_of_Ctrl

Stratum d.f. s.e. cv%

Rep 3 1.508 2.1

Rep.PlotNo 12 5.694 7.8

Analysis of variance (Pyro-res_AR)

Variate: SL%_of_Ctrl

Source of variation d.f. s.s. m.s. v.r. F pr.

Rep stratum 3 0.00 0.00

Rep.PlotNo stratum

R_Eve 4 32000.00 8000.00

Residual 12 0.00 0.00

Total 19 32000.00

Tables of means

Variate: SL%_of_Ctrl

Grand mean 20.00

R_Eve 0 10+10 20 5+5+5+5

0.00 0.00 0.00 0.00

R_Eve UTC

100.00

Standard errors of differences of means

Table R_Eve

rep. 4

d.f. *

s.e.d. 0.000

Least significant differences of means (5% level)

Table R_Eve

rep. 4

d.f. *

l.s.d. *

Stratum standard errors and coefficients of variation

Variate: SL%_of_Ctrl

Stratum d.f. s.e. cv%

Rep 3 0.000 0.0

Rep.PlotNo 12 0.000 0.0

Analysis of variance (Pyro-Res-CU)

Variate: SL%_of_Ctrl

Source of variation d.f. s.s. m.s. v.r. F pr.

Rep stratum 3 430.71 143.57 7.57

Rep.PlotNo stratum

R_Eve 4 6976.63 1744.16 91.93 <.001

Residual 12 227.67 18.97

Total 19 7635.01

Tables of means

Variate: SL%_of_Ctrl

Grand mean 67.44

R_Eve 0 10+10 20 5+5+5+5

42.06 62.55 63.72 68.88

R_Eve UTC

100.00

Standard errors of differences of means

Table R_Eve

rep. 4

d.f. 12

s.e.d. 3.080

Least significant differences of means (5% level)

Table R_Eve

rep. 4

d.f. 12

l.s.d. 6.711

Stratum standard errors and coefficients of variation

Variate: SL%_of_Ctrl

Stratum d.f. s.e. cv%

Rep 3 5.359 7.9

Rep.PlotNo 12 4.356 6.5

Analysis of variance (Pyro-Soil-AR)

Variate: SL%_of_Ctrl

Source of variation d.f. s.s. m.s. v.r. F pr.

Rep stratum 3 115.80 38.60 1.00

Rep.PlotNo stratum

R_Eve 4 30060.30 7515.08 194.69 <.001

Residual 12 463.20 38.60

Total 19 30639.30

Tables of means

Variate: SL%_of_Ctrl

Grand mean 24.1

R_Eve 0 10+10 20 5+5+5+5

20.7 0.0 0.0 0.0

R_Eve UTC

100.0

Standard errors of differences of means

Table R_Eve

rep. 4

d.f. 12

s.e.d. 4.39

Least significant differences of means (5% level)

Table R_Eve

rep. 4

d.f. 12

l.s.d. 9.57

Stratum standard errors and coefficients of variation

Variate: SL%_of_Ctrl

Stratum d.f. s.e. cv%

Rep 3 2.78 11.5

Rep.PlotNo 12 6.21 25.7

Analysis of variance (Pyro-Soil-CU)

Variate: SL%_of_Ctrl

Source of variation d.f. s.s. m.s. v.r. F pr.

Rep stratum 3 1.87 0.62 0.02

Rep.PlotNo stratum

R_Eve 4 11224.46 2806.12 92.45 <.001

Residual 12 364.24 30.35

Total 19 11590.58

Tables of means

Variate: SL%_of_Ctrl

Grand mean 57.7

R_Eve 0 10+10 20 5+5+5+5

66.9 45.3 34.8 41.8

R_Eve UTC

100.0

Standard errors of differences of means

Table R_Eve

rep. 4

d.f. 12

s.e.d. 3.90

Least significant differences of means (5% level)

Table R_Eve

rep. 4

d.f. 12

l.s.d. 8.49

Stratum standard errors and coefficients of variation

Variate: SL%_of_Ctrl

Stratum d.f. s.e. cv%

Rep 3 0.35 0.6

Rep.PlotNo 12 5.51 9.5

Analysis of variance (Tri-Res-AR)

Variate: SL%_of_Ctrl

Source of variation d.f. s.s. m.s. v.r. F pr.

Rep stratum 3 747.7 249.2 2.47

Rep.PlotNo stratum

R_Eve 4 25898.0 6474.5 64.28 <.001

Residual 12 1208.7 100.7

Total 19 27854.4

Tables of means

Variate: SL%_of_Ctrl

Grand mean 34.5

R_Eve 0 10+10 20 5+5+5+5

0.0 8.3 19.9 44.3

R_Eve UTC

100.0

Standard errors of differences of means

Table R_Eve

rep. 4

d.f. 12

s.e.d. 7.10

Least significant differences of means (5% level)

Table R_Eve

rep. 4

d.f. 12

l.s.d. 15.46

Stratum standard errors and coefficients of variation

Variate: SL%_of_Ctrl

Stratum d.f. s.e. cv%

Rep 3 7.06 20.5

Rep.PlotNo 12 10.04 29.1

Analysis of variance (Tri-Res-CU)

Variate: SL%_of_Ctrl

Source of variation d.f. s.s. m.s. v.r. F pr.

Rep stratum 3 751.73 250.58 8.85

Rep.PlotNo stratum

R_Eve 4 1849.44 462.36 16.33 <.001

Residual 12 339.72 28.31

Total 19 2940.88

Tables of means

Variate: SL%_of_Ctrl

Grand mean 84.2

R_Eve 0 10+10 20 5+5+5+5

74.6 76.8 79.0 90.4

R_Eve UTC

100.0

Standard errors of differences of means

Table R_Eve

rep. 4

d.f. 12

s.e.d. 3.76

Least significant differences of means (5% level)

Table R_Eve

rep. 4

d.f. 12

l.s.d. 8.20

Stratum standard errors and coefficients of variation

Variate: SL%_of_Ctrl

Stratum d.f. s.e. cv%

Rep 3 7.08 8.4

Rep.PlotNo 12 5.32 6.3

Analysis of variance (Tri-Soil-AR)

Variate: SL%_of_Ctrl

Source of variation d.f. s.s. m.s. v.r. F pr.

Rep stratum 3 232.79 77.60 0.95

Rep.PlotNo stratum

R_Eve 4 12963.29 3240.82 39.67 <.001

Residual 12 980.27 81.69

Total 19 14176.34

Tables of means

Variate: SL%_of_Ctrl

Grand mean 51.4

R_Eve 0 10+10 20 5+5+5+5

27.2 38.6 40.2 50.9

R_Eve UTC

100.0

Standard errors of differences of means

Table R_Eve

rep. 4

d.f. 12

s.e.d. 6.39

Least significant differences of means (5% level)

Table R_Eve

rep. 4

d.f. 12

l.s.d. 13.92

Stratum standard errors and coefficients of variation

Variate: SL%_of_Ctrl

Stratum d.f. s.e. cv%

Rep 3 3.94 7.7

Rep.PlotNo 12 9.04 17.6

Analysis of variance (Tri-Soil-CU)

Variate: SL%_of_Ctrl

Source of variation d.f. s.s. m.s. v.r. F pr.

Rep stratum 3 41.39 13.80 1.23

Rep.PlotNo stratum

R_Eve 4 1874.06 468.51 41.70 <.001

Residual 12 134.82 11.23

Total 19 2050.27

Tables of means

Variate: SL%_of_Ctrl

Grand mean 81.60

R_Eve 0 10+10 20 5+5+5+5

72.36 81.61 75.84 78.21

R_Eve UTC

100.00

Standard errors of differences of means

Table R_Eve

rep. 4

d.f. 12

s.e.d. 2.370

Least significant differences of means (5% level)

Table R_Eve

rep. 4

d.f. 12

l.s.d. 5.164

Stratum standard errors and coefficients of variation

Variate: SL%_of_Ctrl

Stratum d.f. s.e. cv%

Rep 3 1.661 2.0

Rep.PlotNo 12 3.352 4.1
